# Caudate-anchored cognitive connectivity pursuant to orthostatic hypotension in early Parkinson's disease

**DOI:** 10.1038/s41598-022-26811-w

**Published:** 2022-12-22

**Authors:** Sang-Won Yoo, Seunggyun Ha, Yoon-Sang Oh, Dong-Woo Ryu, Ji-Yeon Yoo, Kwang-Soo Lee, Joong-Seok Kim

**Affiliations:** 1 Department of Neurology, College of Medicine, The Catholic University of Korea, Seoul, Republic of Korea; 2 Division of Nuclear Medicine, Department of Radiology, College of Medicine, The Catholic University of Korea, Seoul, Republic of Korea; 3 Department of Neurology, College of Medicine, The Catholic University of Korea, Seoul St. Mary’s Hospital, 222 Banpo-daero, Seocho-gu, Seoul, 06591 Republic of Korea

**Keywords:** Parkinson's disease, Parkinson's disease

## Abstract

^18^F-Florbetaben is a tracer used to evaluate the metabolic activity of and amyloid accumulation in the brain when measured in early- and late-phase, respectively. The metabolism of neural substrates could be viewed as a network and might be an important factor in cognition. Orthostatic hypotension (OH) might play an indirect moderating role in cognition, and its latent influence could modify the inherent cognitive network. This study aimed to identify changes of cognitive connectivity according to orthostatic stress in patients with early Parkinson’s disease (PD). This study included 104 early PD patients who were evaluated with a head-up tilt-test and^18^F-Florbetaben positron emission tomography (PET). Cognition was assessed with a comprehensive neuropsychological battery that gauged attention/working memory, language, visuospatial, memory, and executive functions. PET images were analyzed visually for amyloid deposits, and early-phase images were normalized to obtain standardized uptake ratios (SUVRs) of pre-specified subregions relevant to specific cognitive domains. The caudate nucleus was referenced and paired to these pre-specified regions. The correlations between SUVRs of these regions were assessed and stratified according to presence of orthostatic hypotension. Among the patients studied, 22 (21.2%) participants had orthostatic hypotension. Nineteen patients (18.3%) were positive for amyloid-β accumulation upon visual analysis. Moderate correlations between the caudate and pre-specified subregions were observed (Spearman’s *rho*, range [0.331–0.545]). Cognition did not differ, but the patterns of correlation were altered when the disease was stratified by presence of orthostatic stress. In conclusion, cognition in early PD responds to hemodynamic stress by adapting its neural connections between regions relevant to cognitive functions.

## Introduction

Orthostatic hypotension (OH) can be observed in early Parkinson’s disease (PD), and it is associated with a more rapid disease progression^1,2^. Previous studies have advocated its adverse effects on cognition^3–5^.

^18^F-Florbetaben (^18^F-FBB) is an amyloid tracer. It has been investigated as a useful biomarker that estimates cerebral perfusion and amyloidosis when measured during the early (post-injection) and delayed phases, respectively^6,7^. The clinical implications of^18^F-FBB on PD with OH (PD + OH) have been investigated, and its early uptake could be interpreted as a sign of metabolic activity^8–10^. Such metabolic neuroimaging has been used to explore the computational brain metabolic connectome^11^, but its links were rarely stratified by PD endophenotypes.

Our previous work argued for the existence of *indirect* moderation of orthostatic blood pressure (BP) change with an inverse association between subcortical atrophy and cognition, and suggested that its latent pathophysiology might be due to dysfunctional connections^12^. Caudate nucleus is involved in cognition, execution, emotion and perceptual functions with somatotopic topography^13,14^. Its dopaminergic denervation in PD also plays an indirect role in cognitive impairment^15^. This idea led to the assumption that the neural network between caudate and cortical substrates in early PD could be shaped by OH and affect cognition that is influenced by subcortical monoamine dysregulation^16,17^.

This study aimed to clarify the latent influence of OH on patterns of cognitive connectivity. Rather than relying on a computational approach, the hypothesis was assessed using a simplified, intuitive schema. Each caudate nucleus was seeded as a reference for each hemisphere and was paired with ipsi-cortical regions relevant to five domains of cognition without inter-hemispheric cross-correlations. It was hypothesized that changes in patterns pursuant to OH would further explain the neurobiological basis of cognition in PD phenotypes.

## Results

Table 1 summarizes the baseline characteristics of the study population. The mean age was 69.9 ± 8.4 years, and 41 (39.4%) were female. The average disease duration was 1.4 ± 1.2 years. The total sum of the Unified Parkinson’s Disease Rating Scale (UPDRS) was 27.6 ± 14.0, with a median H&Y stage of 2.0 (IQR, 0.0). Nineteen PD patients (18.3%) were amyloid-β (Aβ)-positive based on visual analysis of^18^F-FBB PET scans.

**Table 1:** Clinical characteristics.

		PD	PD No-OH	PD + OH	p value
		(n = 104)	(n = 82)	(n = 22)	
Age, year	Mean ± SD	69.9 ± 8.4	69.1 ± 8.9	72.9 ± 5.4	0.01620
Sex, female	n (%)	41 (39.4)	34 (41.5)	7 (31.8)	0.46920
BMI (Kg/m^2^)	Mean ± SD	24.0 ± 2.7	24.0 ± 2.7	23.7 ± 3.0	0.60955
Disease duration, years	Mean ± SD	1.4 ± 1.2	1.4 ± 1.3	1.3 ± 0.9	0.85730
Education, years	Mean ± SD	10.6 ± 4.5	11.2 ± 4.3	8.7 ± 4.6	0.02010
Hypertension	n (%)	55 (52.9)	41 (50.0)	14 (63.6)	0.33741
Diabetes mellitus	n (%)	25 (24.0)	20 (24.4)	5 (22.7)	1.00000
Dyslipidemia	n (%)	52 (50.0)	40 (48.8)	12 (54.5)	0.81071
Non-smoker	n (%)	98 (94.2)	76 (92.7)	22 (100.0)	0.33813
MMSE	Mean ± SD	26.5 ± 3.2	26.8 ± 3.3	25.5 ± 3.0	0.11995
CDR	Median, IQR	0.5 (0.0)	0.5 (0.0)	0.5 (0.0)	0.34319
**UPDRS, total**	Mean ± SD	27.6 ± 14.0	27.0 ± 13.6	29.5 ± 15.5	0.46895
UPDRS Part I	Median, IQR	1.9 ± 1.9	1.8 ± 1.8	2.2 ± 2.0	0.32288
UPDRS Part II	Mean ± SD	6.5 ± 4.6	6.4 ± 4.5	7.0 ± 4.9	0.60338
UPDRS Part III	Mean ± SD	19.2 ± 10.0	19.0 ± 9.8	20.3 ± 10.6	0.58624
H&Y	Median (IQR)	2.0 (0.0)	2.0 (0.0)	2.0 (0.0)	0.44949
Lateralization index	Median (IQR)	0.3 (0.5)	0.3 (0.6)	0.2 (0.3)	0.21439
Mean supine SBP^+^	Mean ± SD	124.7 ± 16.2	122.1 ± 15.8	134.2 ± 14.3	0.00160
Mean supine DBP^+^	Mean ± SD	71.3 ± 8.5	70.7 ± 8.4	73.8 ± 8.2	0.12974
Orthostatic ΔSBP^+^	Mean ± SD	11.0 ± 14.0	5.6 ± 8.5	30.8 ± 12.5	< 0.00001
Orthostatic ΔDBP^+^	Mean ± SD	2.4 ± 7.1	0.1 ± 4.8	11.0 ± 7.5	< 0.00001
Amyloid positivity	n (%)	19 (18.3)	15 (18.3)	4 (18.2)	1.00000

OH, orthostatic hypotension; BMI, body mass index; MMSE, Mini-Mental Status Examination; CDR, Clinical Dementia Rating; H&Y, Hoehn and Yahr; UPDRS, Unified Parkinson’s Disease Rating Scale; SBP, systolic blood pressure; DBP, diastolic blood pressure; SD, standard deviation; IQR, interquartile range.

Group differences were compared using independent or Welch’s t-tests, Mann–Whitney U test or Fisher’s exact test, as appropriate. Statistical significance was defined as a two-tailed p < 0.05.

^+^Blood pressure unit is mmHg.

Twenty-two (21.2%) patients had OH. The disease severity and clinical asymmetry were compared between the groups with and without OH, and they did not differ significantly (Table 1). Global cognition was also not disparate between the two groups (Table 1). The PD + OH patients had a higher average supine BP than did those without OH (PD No-OH *vs*. PD + OH: supine SBP, 122.1 ± 15.8 mmHg *vs.* 134.2 ± 14.3 mmHg, p = 0.00160).

**Figure 1:**
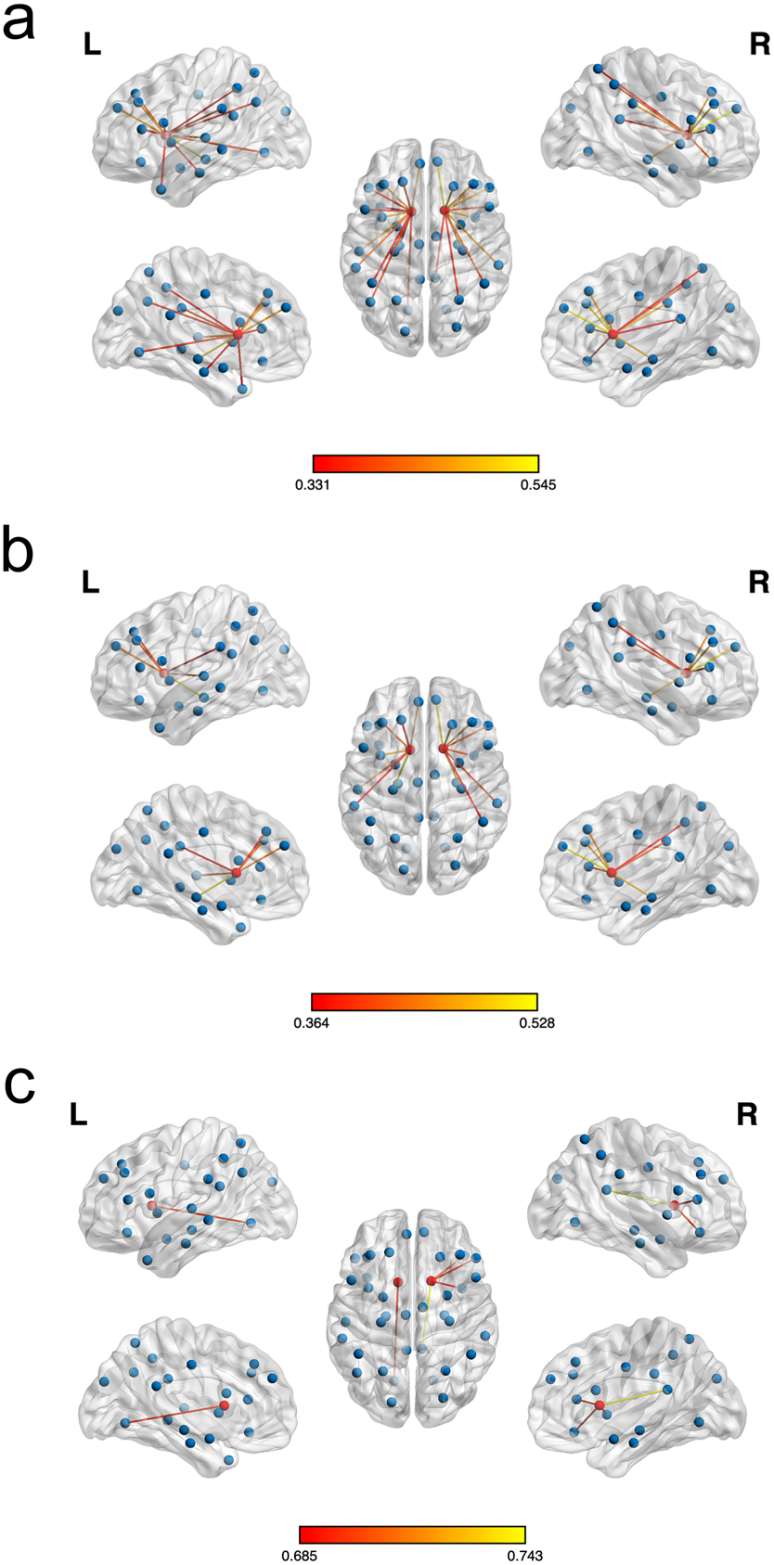
The Spearman correlation coefficients of the entire population (**a**), PD No-OH (**b**) and PD + OH (**c**) are expressed as the edges between the caudate and the ROIs. The degree of correlation is expressed with color; the brighter is the connection, the stronger it is. (**a**) In the analysis of all patients studied, the caudate nucleus maintained a moderate degree of association with the prefrontal areas; insula; hippocampus; parahippocampus; posterior cingulate gyrus; lingual gyrus; superior/inferior parietal gyrus; and the supramarginal, angular, superior temporal, and temporal pole areas. (**b**) In PD No-OH, the caudate-refenced associations with the dorsolateral and medial superior frontal, middle/inferior frontal gyrus, insula, hippocampus, inferior parietal gyrus, supramarginal gyrus, and superior temporal gyrus remained significant. However, the number of significant connections was reduced compared to the whole population, and degrees of correlation remained moderate. (**c**) PD + OH patients manifested different traits. Most of the observed connections of the left caudate nucleus lost their significance, except the correlation with the left lingual gyrus. A few links between the right caudate and right inferior frontal gyrus, insula, and posterior cingulate gyrus remained significant. Of note, the strength of the remaining connections increased. The figures were made with BrainNET Viewer, a graphical user interface (version 1.7; http//www.nitrc.org/projects/bnv).

Table 2 and Fig. 1 encapsulate the distinct patterns of caudate-centered cognitive connectivity across pre-specified ROIs relevant to five domains of cognition. As a whole, the caudate nucleus maintained moderate associations with the prefrontal areas; insula; hippocampus; parahippocampus; posterior cingulate gyrus; lingual gyrus; superior/inferior parietal gyrus; and supramarginal, angular, superior temporal, and temporal pole areas (Spearman partial correlation coefficient, *rho*, range [0.331–0.545]).

**Table 2:** Spearman partial correlation coefficients of prespecified subregions.

Subregion	PD (n = 104)				PD No-OH (n = 82)				PD + OH (n = 22)			
	Left caudate	*p* value	Right caudate	*p* value	Left caudate	*p* value	Right caudate	*p* value	Left caudate	*p* value	Right caudate	*p* value
Superior frontal gyrus, dorsolateral	0.410**	0.00002	0.468**	< 0.00001	0.391**	0.00034	0.452**	0.00003	0.514	0.02035	0.520	0.01879
Middle frontal gyrus	0.456**	< 0.00001	0.528**	< 0.00001	0.415**	0.00013	0.480**	< 0.00001	0.600	0.00513	0.610	0.00431
Inferior frontal gyrus, opercular part	0.359**	0.00021	0.400**	0.00003	0.352	0.00136	0.332	0.00261	0.329	0.15711	0.625	0.00320
Inferior frontal gyrus, triangular part	0.355**	0.00025	0.476**	< 0.00001	0.325	0.00329	0.410**	0.00016	0.597	0.00540	0.692**	0.00072
Inferior frontal gyrus, pars orbitalis	0.304	0.00192	0.412**	0.00002	0.286	0.01008	0.362	0.00095	0.394	0.08597	0.699**	0.00060
Superior frontal gyrus, medial	0.462**	< 0.00001	0.545**	< 0.00001	0.444**	0.00004	0.513**	< 0.00001	0.602	0.00495	0.651	0.00189
Insula	0.495**	< 0.00001	0.455**	< 0.00001	0.485**	< 0.00001	0.394**	0.00030	0.545	0.01303	0.685**	0.00086
Middle cingulate and paracingulate gyrus	0.177	0.07514	0.236	0.01706	0.085	0.45216	0.143	0.20576	0.512	0.02113	0.549	0.01214
Posterior cingulate gyrus	0.259	0.00851	0.334**	0.00061	0.168	0.13522	0.243	0.03017	0.593	0.00583	0.743**	0.00017
Hippocampus	0.517**	< 0.00001	0.463**	< 0.00001	0.528**	< 0.00001	0.480**	< 0.00001	0.631	0.00286	0.534	0.0152
Parahippocampus	0.331**	0.00068	0.144	0.14807	0.286	0.01015	0.095	0.40148	0.511	0.02131	0.442	0.05091
Amygdala	0.229	0.02034	0.134	0.17810	0.156	0.16613	0.094	0.40477	0.569	0.00883	0.416	0.06838
Lingual gyrus	0.369**	0.00013	0.304	0.00191	0.293	0.00826	0.245	0.02829	0.695**	0.00066	0.530	0.01625
Superior occipital gyrus	0.174	0.07982	0.261	0.00812	0.105	0.35213	0.186	0.09898	0.361	0.11803	0.522	0.01816
Superior parietal gyrus	0.307	0.00169	0.349**	0.00032	0.267	0.01648	0.303	0.00638	0.367	0.11121	0.484	0.03077
Inferior parietal gyrus*	0.334**	0.00061	0.399**	0.00003	0.302	0.00646	0.375**	0.00060	0.397	0.08288	0.451	0.04596
Supramarginal gyrus	0.405**	0.00002	0.465**	< 0.00001	0.364**	0.00090	0.405**	0.00019	0.623	0.00335	0.634	0.00270
Angular gyrus	0.363**	0.00018	–	–	0.356	0.00117	–	–	0.460	0.04124	–	–
Superior temporal gyrus	0.458**	< 0.00001	–	–	0.434**	0.00006	–	–	0.574	0.00809	–	–
Middle temporal gyrus	0.280	0.00437	–	–	0.255	0.02241	–	–	0.513	0.02057	–	–
Temporal pole: middle temporal gyrus	0.334**	0.00061	–	–	0.321	0.00375	–	–	0.241	0.30603	–	–

OH, orthostatic hypotension.

*Supramarginal and angular gyrus are excluded.

Spearman partial correlation coefficients, partialized by age and disease duration, were calculated. Each caudate (seed ROI) was only correlated to ipsilateral subregions, i.e., left caudate’s correlations with left hemispheric subregions.

**Coefficients whose *p* value < 0.00094 (Bonferroni correction threshold: < 0.05/53) were selected for further analyses.

The pattern of associations changed when the study population was divided into PD No-OH and PD + OH groups. In the PD + OH patients, most of the observed connections of the left caudate nucleus lost their significance, except that with the left lingual gyrus (*rho*, 0.695, p = 0.00066). A few links between the right caudate and right inferior frontal gyrus, insula, and posterior cingulate gyrus retained their significance. Of note, the strength of the remaining connections increased (*rho*, range [0.685–0.743]).

The PD No-OH patients manifested different traits compared to PD + OH group. Although the caudate-referenced associations with the dorsolateral and medial superior frontal, middle/inferior frontal gyrus, insula, hippocampus, inferior parietal gyrus, supramarginal gyrus, and superior temporal gyrus remained significant, the number of significant connections was reduced compared to that of the whole population. The degrees of correlation remained within a moderate range (*rho*, range [0.364–0.528]).

The strength of connectivity between corresponding correlations was compared (Table 3). The inter-hemispheric comparisons of two dependent correlations among the entire study population and its sub-populations did not reveal any significant differences; there was no difference between left and right connectivity. The independent topology-matched correlations of each hemisphere in both PD No-OH and PD + OH patients were assessed to identify their differences. Tests of significance failed to show any disparity except of the posterior cingulate gyrus and the left lingual gyrus when multiple comparisons were not adjusted.

**Table 3:** Intra/interhemispheric regional differences of Spearman partial correlation coefficients.

Subregion	Within-group comparison of inter-hemispheric correlations						Between-group comparison of ipsi-hemispheric correlations			
	PD (n = 104)		PD No-OH (n = 82)		PD + OH (n = 22)		No-OH *vs.* OH			
	Steiger's z test	*p* value	Steiger's z test	*p* value	Steiger's z test	*p* value	Fisher'z test (*L*)	*p* value	Fisher'z test (*R*)	*p* value
Superior frontal gyrus, dorsolateral	− 1.026	0.30491	− 0.937	0.34889	− 0.049	0.96090	0.607	0.54367	0.349	0.72723
Middle frontal gyrus	− 1.340	0.18025	− 1.010	0.31235	− 0.100	0.92031	0.984	0.32496	0.728	0.46681
Inferior frontal gyrus, opercular part	− 0.587	0.55718	0.248	0.80418	− 1.877	0.06052	0.102	0.91890	1.519	0.12880
Inferior frontal gyrus, triangular part	− 1.751	0.08002	− 1.082	0.27939	− 0.723	0.46945	1.375	0.16924	1.629	0.10337
Inferior frontal gyrus, pars orbitalis	− 1.488	0.13684	− 0.952	0.34087	− 1.789	0.07364	0.479	0.63214	1.903	0.05709
Superior frontal gyrus, medial	− 1.709	0.08745	1.231	0.21846	− 0.502	0.61549	0.857	0.39124	0.823	0.41062
Insula	0.680	0.49669	1.316	0.18827	− 1.297	0.19474	0.320	0.74905	1.651	0.09867
Middle cingulate and paracingulate gyrus	− 0.942	0.34635	− 0.806	0.42043	− 0.325	0.74531	1.879	0.06019	1.851	0.06417
Posterior cingulate gyrus	− 1.095	0.27342	− 0.946	0.34437	− 1.229	0.21904	2.006	0.04481	2.775	0.00551
Hippocampus	0.960	0.33713	0.748	0.45446	0.919	0.35826	0.609	0.54227	0.285	0.77589
Parahippocampus	2.652	0.00800	2.354	0.01856	0.402	0.68767	1.056	0.29088	1.485	0.13756
Amygdala	1.201	0.22960	0.693	0.48827	0.995	0.31964	1.913	0.05577	1.364	0.17252
Lingual gyrus	1.038	0.29907	0.629	0.52916	1.643	0.10034	2.175	0.02964	1.331	0.18324
Superior occipital gyrus	− 1.195	0.23199	− 0.984	0.32525	− 1.087	0.27690	1.067	0.28596	1.530	0.12606
Superior parietal gyrus	− 0.622	0.53427	− 0.466	0.64142	− 0.648	0.51723	0.436	0.66308	0.843	0.39929
Inferior parietal gyrus*	− 0.783	0.43379	− 0.756	0.44954	− 0.323	0.74691	0.424	0.67150	0.359	0.71961
Supramarginal gyrus	–	–	–	–	–	–	1.364	0.17272	–	–
Angular gyrus	–	–	–	–	–	–	0.489	0.62466	–	–
Superior temporal gyrus	–	–	–	–	–	–	0.738	0.46032	–	–
Middle temporal gyrus	–	–	–	–	–	–	1.198	0.23103	–	–
Temporal pole: middle temporal gyrus	–	–	–	–	–	–	0.340	0.73371	-	-

Steiger’z test was performed to compare two dependent correlations between topology-matched subregions of each hemisphere within a group, i.e., the associations of left caudate and left dorsolateral superior frontal gyrus (left hemisphere) compared to the associations of right caudate and right dorsolateral superior frontal gyrus (right hemisphere) within the same groups. Calculations were not performed in such instances where comparisons of corresponding subregions were not feasible as the language domain, e.g., the relation between left caudate and left supramarginal gyrus did not have an equivalent relation between right caudate and right supramarginal gyrus.

Fisher’z test was utilized to evaluate the *between-group* difference between two independent correlations from corresponding hemisphere of No-OH *vs.* OH. For example, the association of left caudate and left dorsolateral superior frontal gyrus (left hemisphere) from PD No-OH was compared to the association of left caudate and left dorsolateral superior frontal gyrus (left hemisphere) from PD OH, Fisher’z test (*L*).

None survived multiple comparison adjustments.

*Supramarginal and angular gyrus are excluded.

The z-scores of each cognitive subtest were compared between PD No-OH and PD + OH patients (Table 4). These comparisons did not survive multiple comparison adjustments. When multiple comparisons were not corrected, the SVLT recognition z-scores were lower in PD + OH patients than in PD No-OH patients (PD No-OH *vs*. PD + OH: SVLT-E: Recognition, − 0.3 ± 0.1 *vs.* − 0.9 ± 0.3, p = 0.04233).

**Table 4:** Between-group differences of global cognition and its subdomains.

Global cognition	PD No-OH	PD + OH	p value
	(n = 82)	(n = 22)	
Global z-score^1^	− 0.5 ± 0.1	− 0.5 ± 0.2	0.73664
**Cognitive subdomains**			
Attention/Working memory			
Digit Span Forward	0.1 ± 0.1	0.1 ± 0.2	0.88224
K-CWST	− 0.6 ± 0.1	− 0.8 ± 0.2	0.46739
Attention/working memory score^2^	− 0.3 ± 0.1	− 0.4 ± 0.2	0.55358
Executive			
Digit Span Backward	− 0.4 ± 0.1	− 0.1 ± 0.2	0.30577
COWAT: Phonemic	− 0.5 ± 0.1	− 0.1 ± 0.2	0.10321
Executive score^3^	− 0.5 ± 0.1	− 0.1 ± 0.2	0.10947
Language			
K− BNT	− 0.3 ± 0.2	− 0.4 ± 0.4	0.86888
Verbal memory			
SVLT-E: Immediate recall	− 0.7 ± 0.1	− 1.1 ± 0.2	0.07705
SVLT-E: Delayed recall	− 0.7 ± 0.1	− 0.6 ± 0.2	0.65975
SVLT-E: Recognition	− 0.3 ± 0.1	− 0.9 ± 0.3	0.04233
Visual memory			
RCFT: Immediate recall	− 0.5 ± 0.1	− 0.2 ± 0.2	0.19451
RCFT: Delayed recall	− 0.6 ± 0.1	− 0.3 ± 0.2	0.31181
RCFT: Recognition	− 0.5 ± 0.1	− 1.0 ± 0.3	0.12076
Memory score^4^	− 0.6 ± 0.1	− 0.5 ± 0.2	0.37477
Visuospatial			
RCFT	− 1.0 ± 0.2	− 1.0 ± 0.4	0.94539

Scores of neuropsychological assessments are presented as means ± standard error (SE).

Between-group disparities of cognitive tests were investigated by analysis of covariance (ANCOVA), adjusted for age, disease duration, and education years. Statistical significance was defined as a two-tailed p-value < 0.05.

K-CWST, Korean-Color Word Stroop Test; COWAT, Controlled Oral Word Association Test; K-BNT, Korean-Boston Naming Test; SVLT-E, Seoul Verbal Learning Test-Elderly’s version; RCFT, Rey Complex Figure Test.

^1^Average z-scores of attention/working memory score, executive score, K-BNT, memory score, and RCFT.

^2^Average z-scores of Digit Span Forward, K-CWST.

^3^Average z-scores of Digit Span Backward, COWAT: Phonemic.

^4^Average z-scores of SVLT-E: Delayed recall and RCFT: Delayed recall.

## Discussion

In de novo*,* early and drug-naïve PD, distinct subclinical patterns of caudate-seeded connectivity were observed between PD No-OH and PD + OH patients. The caudate nucleus of each hemisphere was coupled with prespecified regions relevant to cognition. The patterned correlations were changed by BP instability, but these alterations did not culminate into between-group differences in cognition. This finding indicates intrinsic cognitive resilience in early PD.

This cohort enrolled early PD patients with mild parkinsonism. The prevalence of OH (21.2%) was comparable to that of early PD in another study^2^. PD + OH patients were slightly older and had a higher supine systolic BP compared to PD No-OH patients, as reported by previous studies^9,18^. This suggests that PD + OH patients could have suffered greater cerebral hemodynamic insult due to wider BP fluctuations^19^. Each caudate nucleus was significantly correlated to multiple ipsilateral regions. Both sides similarly contributed to a particular cognitive domain unless defined to be lateralized as the language domain (i.e., the left angular or superior/middle temporal gyrus was only selected for its function).

Caudate nucleus participates in information processing as a hub that is part of distributed networks involved in cognition, execution, emotion and perceptual functions with somatotopic topography^13,14^. It was also postulated that caudate might play an indirect role in cognitive impairment of PD whose dopaminergic input to caudate degenerated^15,17^. Widespread connections of caudate nucleus to the functional compartments of the brain and its influence on cortical functions led to presuppose the stratified caudate-focused connectivity with orthostatic stress.

Cognitive domains evaluated by compressive neuropsychological tests are comprised of multiple specialized cortical areas paralleled as subnetworks within brain. Particular domains such as language and attention imply hemispheric dominance (the left for language *vs.* the right for visuospatial attention; detailed in supplementary Table 1)^20,21^. Multiple correlation analyses investigated the adjoining role of caudate and its bonds with organized cortical areas, focusing on the changes of connectivity by orthostatic challenge. Analyses without cross-correlations were executed as only a small fraction of neurons project to the contralateral hemisphere, mostly homotopic connections^22^. Inclusion of clinically irrelevant cross-correlations might result in unexpected multiple comparison problems; thus a priori regions were selected.

In this cohort, two main findings were observed. There were no differences in cognition between PD No-OH and PD + OH patients, but the caudate-seeded connectivity revealed subclinical changes according to orthostatic vulnerability. The resulting null consequence of clinical comparison and the alternative adaptive variation of connections support the *indirect* influence of OH^12^. These outcomes are compatible with the thesis to specify the latent neurobiological role of hemodynamic challenge on cognitive connectivity; there was no direct *difference* in clinometrics, but there was *moderation* of the underlying neural mechanism.

Our results suggest that different networks may be inherent in distinct PD subtypes. This distinction could explain the indistinguishable difference in cognition between PD No-OH and PD + OH patients which may represent the compensatory cognitive resiliency observed in early PD^23,24^. The number of significant connections was reduced, but the degrees increased in PD + OH compared to those without OH. Compensation for orthostatic stress was discussed in a previous study^9^, and it could be integrated in the form of cognitive network expression^23^. The enrolled patients were in the early stage of PD, which might have produced a subthreshold for clinical differences.

Aging and education level were argued for their roles in cognitive reserve as diluters of pre-existing network efficiency within individuals^23^. As PD + OH were slightly older and less educated, they could have influenced for the changes of pattern in connectivity. On the other hand, the intensified degrees among weakened connections could be explained as the compensation mechanism by the intrinsic hemodynamic stress of PD itself.

There were no significant gaps in the intensities of the associations between the left and right hemispheres in any sub-population. When PD No-OH and PD + OH patients were compared by hemisphere to the corresponding hemisphere of the other group, no relationship remained after multiple comparison corrections. This finding signified that both hemispheres equally contributed to cognition, and the effects of OH were not biased.

Aβ has been observed in PD^25^. Its presence was also observed in our cohort. The prevalence was similar between PD No-OH and PD + OH patients; thus, its involvement in pattern stratification was not considered in the analyses.

This study had several limitations. First, early PD was investigated using a cross-sectional design. Further longitudinal studies that include different stages of PD are required to observe any changes in the patterns of correlations and to consolidate the cognitive resilience theory in PD. Moreover, because early PD was enrolled and followed for a relatively short period of time, there might be a chance that other synucleinopathies such as multiple system atrophy had been recruited. Second, we did not investigate the connectome based on topology of delayed^18^F-FBB accumulations. In this study, we obtained^18^F-FBB PET imaging, which enables simultaneous interpretation of cerebral perfusion and the influence of Aβ toxicity at the same time. The effects of amyloidogenic synaptic toxicity on networks was investigated previously^26,27^. It would be very informative to define the cognitive connectomes of PD brain in combination with perfusion/metabolism interpretation. However, the number of amyloid-positive PD on visual analysis was too small for further analysis. Future study with sufficient sample size to search interaction between amyloid deposit and perfusion is warranted. Third, the design of this study was oversimplified in that it did not incorporate fully the complex network of the brain. Computational models have been employed in other studies, and the assumption of this cohort needs to be tested^28,29^. However, the schema presented in our research is understandable and acceptable in that a clinically hypothesis-driven approach was applied instead of extensive computations. Fourth, one could argue that as early PD is characterized by asymmetric manifestations, it would be more appropriate to perform analyses based on the laterality. In this study, the size of laterality was found to be small, and insignificant when stratified by the orthostatic stress. Thus, correlation analyses of prespecified cortical regions pertinent to established laterality of cortical functions seemed applicable. Lastly, the results might vary depending on the employed normalization method. Diverse methodology needs to be applied and results be replicated in future studies.

In sum, the current findings suggest that underlying neural mechanisms, such as cognitive connectivity, can endure hemodynamic stress to maintain cognition in early PD. This cognitive resiliency should be examined in future studies enrolling different subtypes of PD and applying comprehensive network models.

## Methods

### Participants

This study was approved by the Institutional Review Board of Seoul St. Mary’s Hospital, and all participants provided written informed consent to participate. All experiments were performed in accordance with relevant guidelines and regulations.

We enrolled 104 drug-naïve, de novo PD patients diagnosed between December 2018 and August 2020. The participants had been diagnosed with idiopathic PD according to the criteria of the United Kingdom PD Society Brain Bank^30^. All participants were evaluated with positron emission tomography (PET) using^18^F-*N*-(3-fluoropropyl)-2β-carbon ethoxy-3β-(4-iodophenyl) nortropane, and they demonstrated decreased presynaptic dopamine transporter uptake, predominantly in the posterior striatum. They were followed with average duration of 20.0 ± 8.8 months and reaffirmed as PD by the two neurologists (S.-W. Y., J.-S. K.). Demographics including age; sex; disease duration; body mass index; education status; smoking status; and history of hypertension, diabetes mellitus, or dyslipidemia were obtained by interviews or medical records. Patients with the following indications were excluded: (1) symptoms or signs of atypical PD and secondary Parkinsonism; (2) documentation of atrial fibrillation during the head-up tilt test; and (3) history of autonomic neuropathy.

### Disease severity and lateralization index

Disease severity was assessed with the Unified Parkinson’s Disease Rating Scale (UPDRS) and Hoehn and Yahr (H&Y) stages. Clinical asymmetry of symptom severity was elicited from UPDRS, part III score. To assume lateralization index, the absolute difference between total sum of motor scores from each side (right *vs.* left) was divided by the sum of both sides^31^. Closer the ratio to one, the more asymmetric it signified.

### Neuropsychological evaluation

The neuropsychological evaluations were performed by experienced psychologists who were blinded to patient clinical and neuroimaging data. Patient cognitive status was obtained from interviews with caregivers. Five domains of cognition were assessed using a comprehensive neuropsychological battery^32–34^. Attention and working memory were examined with the Digit Span Forward Test and the Korean-Color Word Stroop Test (K-CWST). The executive domain was tested using the Digit Span Backward Test and the Controlled Oral Word Association Test (COWAT). Language and visuospatial domains were assessed by the Korean-Boston Naming Test and the Rey Complex Figure Test (RCFT), respectively. Verbal and visual memory domains were investigated with the Seoul Verbal Learning Test (SVLT) and the RCFT, respectively. Memory domain was divided further into immediate/delayed recall and recognition. Each quantifiable neuropsychological test score was converted into a standardized z-score based on age-, sex-, and education-specific norms. The z-scores of > 1 subtests within a particular domain were averaged to represent the domain, except memory domain, for which the z-scores of delayed recall from the verbal and visual domains were averaged. Cognition was investigated by the Mini-Mental Status Examination, the Clinical Dementia Rating (CDR), and was also rated with the average z-score of attention/working memory, executive and memory scores, language score, and visuospatial function score.

### Head-up tilt test

All participants were fully rested before the procedure. Continuous electrocardiograph leads and non-invasive BP monitoring equipment were applied to the patients (YM6000, Mediana Tech, Redmond, WA, USA). A supine position was maintained for 20 min during which the BP and heart rate were measured every 5 min before tilting to 60 degrees (ENRAF NONIUS, Rotterdam, Netherlands). The same measurements were recorded after 0, 3, 5, 10, 15, and 20 min in the tilted position.

The supine BP at 0 min was excluded, and the average supine systolic and diastolic BPs were calculated from the measurements obtained at 5, 10, 15, and 20 min. The lowest systolic and diastolic BP measurements after 3 or 5 min in the tilted position were selected, and the orthostatic BP changes in systole (ΔSBP) and diastole (ΔDBP) were calculated. When the average supine BP readings were ≥ 140/90 mmHg, ΔSBP and/or ΔDBP ≥ 30/15 mmHg within 5 min were applied to define OH; otherwise, ΔSBP and/or ΔDBP ≥ 20/10 mmHg were assumed^35,36^.

### ^18^F-Florbetaben positron emission tomography imaging acquisition, processing, and interpretation

Dual-phase^18^F-FBB PET-computed tomography (PET-CT) images were acquired using a dedicated PET-CT scanner (Discovery PET-CT 710, General Electric Healthcare, Waukesha, WI, USA). Early-phase PET data were obtained 10 min after intravenous injection of 296 MBq of^18^F-FBB. Ninety minutes after^18^F-FBB injection, delayed-phase PET data were obtained for 20 min. A low-dose CT scan for attenuation correction was obtained immediately before PET imaging. Both early and delayed static PET data were reconstructed using a fully three-dimensional ordered subset-expectation maximization algorithm (VUE Point HD) with 4 iterations and 16 subsets, with a 3-mm Gaussian filter and a 256 × 256 matrix size. Individual structural T1 magnetic resonance imaging (MRI) acquisitions were used for spatial normalization of the PET data.

Statistical Parametric Mapping software, version 8 (University College of London, London, UK, www.fil.ion.ucl.ac.uk/spm) was used for PET image preprocessing. Individual PET images were co-registered to the corresponding T1 MRI images using a rigid transformation. The individual MRI scans were normalized into Montreal Neurological Institute (MNI) space via the T1 MRI template using nonlinear registration. The Automated Anatomical Atlas 3 (AAL3), which consists of 166 regions-of-interest (ROIs), was used to parcellate the ROIs. The entire cerebellum as defined by an atlas from the Centiloid Project was chosen as the reference to calculate the standardized-uptake-value-ratio (SUVR) for semi-quantification of perfusion from early-phase^18^F-FBB PET images. The cerebellar gray template presented by the Centiloid Project was chosen as a reference ROI to determine the SUVR for semi-quantification of amyloid β plaque from the delayed-phase^18^F-FBB PET images^37^. Delayed-phase^18^F-FBB PET imaging was interpreted visually by a nuclear medicine physician (S.H., a co-author of this study) as either positive or negative based on regional^18^F-FBB uptake assessment of the lateral temporal cortex, frontal cortex, posterior cingulate cortex/precuneus, and parietal cortex^38^.

### Regions of interest and correlation analyses

Among the parcellated regions of the AAL atlas, 24 related to five cognitive domains were pre-specified (Supplementary Table 1)^17,21^. The SUVRs of early-phase^18^F-FBB PET images for particular ROIs were used in the correlation analyses. Each caudate nucleus was chosen as a seed region for intra-hemispheric correlation analyses with the ipsilateral pre-specified regions. The caudate nucleus was selected as the anchor for the basal ganglionic cognitive subterritory (Supplementary Fig. 1)^39^. Crossed associations between one side of the caudate and contralateral ROIs were not evaluated. However, inter-hemispheric comparisons of dependent and/or independent correlations of corresponding regions were examined. For example, the correlation of the left caudate and left dorsolateral superior frontal gyrus was assumed to be dependent on the association of the right caudate and right dorsolateral superior frontal gyrus *within* the same population. The correlation of the left caudate and left dorsolateral superior frontal gyrus in one group was assumed to be independent of the association between the left caudate and left dorsolateral superior frontal gyrus of *another* group.

### Statistical analyses

All statistical analyses were performed with jamovi software (version 2.0, Retrieved from https://www.jamovi.org, 2021) and the R (version 4.0, Retrieved from https://cran.r-project.org/, 2021) *car*, *emmeans,* and *psych* packages (Retrieved from https://cran.r-project.org/package=car, https://cran.r-project.org/package=emmeans, and https://cran.r-project.org/package=psych). Descriptive analyses and independent or Welch’s t-test, Mann–Whitney U test, or Fisher’s exact test were applied as appropriate to describe the baseline characteristics of the population. Caudate-seeded, pairwise Spearman partial correlations that had been partialized by age and disease duration were performed to obtain the correlation coefficients between pre-specified ROIs (Supplementary Table 2). In total, 53 correlation analyses were executed.

Steiger’s z tests were utilized to discern *within-group* inter-hemispheric differences of the correlation coefficients of corresponding ROIs (i.e., to test the significance between two dependent correlations)^40^. *Between-group* comparisons of each hemispheric correlation coefficient were investigated by Fisher’s z test, a test of significance between two independent correlations.

The cognition of subgroups was evaluated by analysis of covariance, adjusted for age, disease duration, and years of education. Multiple comparisons were controlled using a Bonferroni correction at a defined significant two-tailed p-value < 0.05.

## Supplementary Information

Supplementary Information.

## Data Availability

Anonymized data generated during the current study are available from the corresponding author on reasonable request from individuals affiliated with research or health care institutions.
