# Supplementary material for: Caudate-anchored cognitive connectivity pursuant to orthostatic hypotension in early Parkinson's disease
Source: Sci Rep. 2022 Dec 22;12:22161. doi: 10.1038/s41598-022-26811-w (PMC9780335; doi:10.1038/s41598-022-26811-w)
Supplement: Supplementary file 1 — Supplementary Information. [file 41598_2022_26811_MOESM1_ESM.docx]

**Supplementary Information for the manuscript:**

**Caudate-anchored cognitive connectivity pursuant to orthostatic hypotension in early Parkinson disease**

Sang-Won Yoo, Seunggyun Ha, Yoon-Sang Oh, Dong-Woo Ryu, Ji-Yeon Yoo, Kwang-Soo Lee, and Joong-Seok Kim


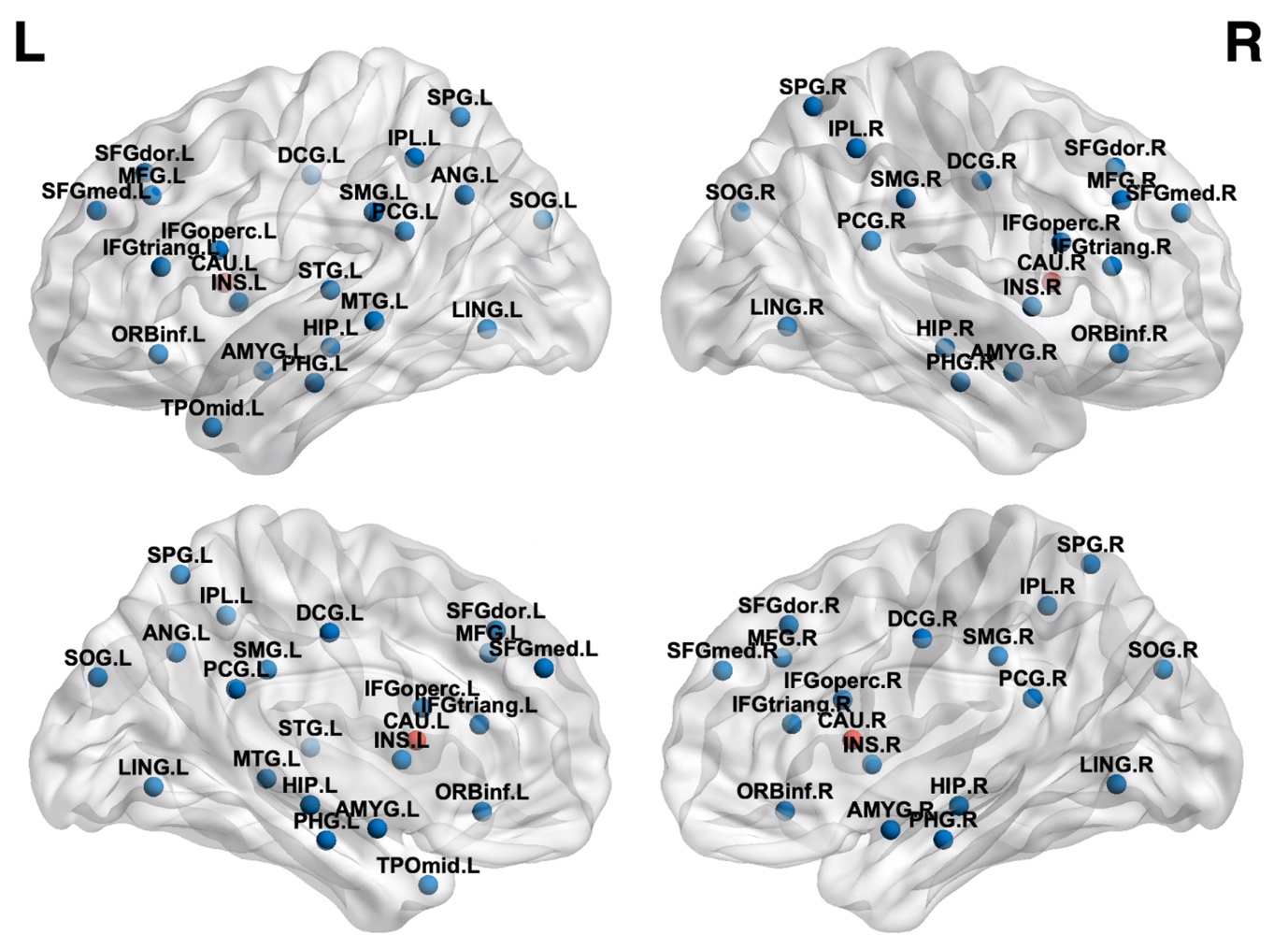
**Supplementary Figure 1**. The prespecified areas of cognitive domains.

Nodes represent the prespecified regions of interest (ROIs).

Red node, caudate nucleus (seed ROI); Blue node, pre-specified ROIs paired to the seed.

L, left; R, right; SFGdor, dorsolateral superior frontal gyrus; MFG, middle frontal gyrus; IFGoperc, inferior frontal gyrus, opercular part; IFGtriang, inferior frontal gyrus, triangular part; ORBinf, inferior frontal gyrus, orbital part; SFGmed, medial superior frontal gyrus; INS, insula; DCG, middle cingulate and paracingulate gyrus; PCG, posterior cingulate gyrus; HIP, hippocampus; PHG, parahippocampus; AMYG, amygdala; LING, lingual gyrus; SOG, superior occipital gyrus; SPG, superior parietal gyrus; IPL, inferior parietal gyrus; SMG, supramarginal gyrus; ANG, angular gyrus; CAU, caudate; STG, superior temporal gyrus; MTG, middle temporal gyrus; TPOmid, temporal pole: middle temporal gyrus.

The figures were made with BrainNET Viewer, a graphical user interface (version 1.7; http//www.nitrc.org/projects/bnv).

Supplementary Table 1. Prespecified areas of cognitive domains

| Domain/function |  | AAL atlas anatomical areas | Abbreviations |
| --- | --- | --- | --- |
| Attention/working memory domain | Dorsolateral prefrontal cortex | Dorsolateral superior frontal gyrus | SFGdor |
|  |  | Middle frontal gyrus | MFG |
|  | Right ventrolateral prefrontal cortex | Right inferior frontal gyrus |  |
|  |  | opercular part | IFGoperc. R |
|  |  | triangular part | IFGtriang. R |
|  |  | orbital part | ORBinf. R |
|  | Posterior parietal cortex | Superior parietal gyrus | SPG |
|  |  | Inferior parietal gyrus (supramarginal and angular gyrus excluded) | IPL |
|  |  | Right supramarginal gyrus | SMG. R |
|  | Medial prefrontal cortex | Medial superior frontal gyrus | SFGmed |
|  |  | Middle cingulate and paracingulate gyrus | DCG |
|  | Insula | Insula | INS |
| Frontal/Executive domain | Dorsolateral prefrontal cortex | Dorsolateral superior frontal gyrus | SFGdor |
|  |  | Middle frontal gyrus | MFG |
|  | Ventrolateral prefrontal cortex | Inferior frontal gyrus |  |
|  |  | opercular part | IFGoperc |
|  |  | triangular part | IFGtriang |
|  |  | orbital part | ORBinf |
|  | Insula | Insula | INS |
|  | Subcortical deep nuclei | Caudate | CAU |
|  |  | Amygdala | AMYG |
| Memory domain | Limbic system | Hippocampus | HIP |
|  |  | Parahippocampus | PHG |
|  |  | Amygdala | AMYG |
|  |  | Posterior cingulate gyrus | PCG |
|  | Retrosplenial cortex | Lingual gyrus | LING |
| Language domain | Wernicke area | Left supramarginal gyrus | SMG. L |
|  |  | Left angular gyrus | ANG. L |
|  |  | Left superior temporal gyrus | STG. L |
|  |  | Left middle temporal gyrus | MTG. L |
|  |  | Left temporal pole: middle temporal gyrus | TPOmid. L |
|  | Broca area | Left inferior frontal gyrus |  |
|  |  | opercular part | IFGoperc. L |
|  |  | triangular part | IFGtriang. L |
| Visuospatial domain |  | Superior occipital gyrus | SOG |
|  |  | Superior parietal gyrus | SPG |

Cognitive domains comprise of interconnected cortical regions as the followings:

1) The anatomical substrates of memory domain are hippocampus with surrounding medial aspects of temporal lobe (parahippocampus, amygdala), and posterior cingulate and retrosplenial cortex(lingual gyrus) as part of circuit of Papez.

2) Attention/working memory domain involves widespread cortical areas, which also requires executive control. It consists of dorsal and ventral attention network that is represented bilaterally and lateralized to the right hemisphere, respectively. Dorsal attention network includes area of dorsolateral prefrontal cortex (dorsolateral superior and middle frontal gyrus) and posterior parietal cortex (superior and inferior parietal lobules). Ventral attention network is consisted of right ventrolateral prefrontal cortex (inferior frontal gyrus) and right temporoparietal cortex (supramarginal gyrus). Executive control of attention involves midcingulate cortex, medial prefrontal cortex (superior frontal gyrus), and anterior insula.

3) Executive domain shares overlapping cortical regions with attention/working memory domain., particularly lateral prefrontal cortex.

4) Language domain is lateralized to the left hemisphere, and posterior and anterior language systems account for different language funcitions. Posterior system responds to receptive language that involves left temporoparietal areas (superior, middle temporal gyrus/temporal pole, supramarginal gyrus, angular gyrus). Anterior system is responsible for expressive language that involves left inferior frontal gyrus.

5) Visuospatial domain is related to cortical areas of superior occipital and parietal gyrus that convey spatial information (*dorsal stream*).

Supplementary Table 2. Individual hemispheric correlation linkage between ipsilateral caudate nucleus (seed) and prespecified subregions

| Domain/function |  | Abbreviations |  | Left caudate | Right caudate |
| --- | --- | --- | --- | --- | --- |
| Attention/working memory domain | Dorsolateral prefrontal cortex | SFGdor |  | O | O |
|  |  | MFG |  | O | O |
|  | Right ventrolateral prefrontal cortex |  |  |  |  |
|  |  | IFGoperc. R |  | - | O |
|  |  | IFGtriang. R |  | - | O |
|  |  | ORBinf. R |  | - | O |
|  | Posterior parietal cortex | SPG |  | O | O |
|  |  | IPL |  | O | O |
|  |  | SMG. R |  | - | O |
|  | Medial prefrontal cortex | SFGmed |  | O | O |
|  |  | DCG |  | O | O |
|  | Insula | INS |  | O | O |
| Frontal/Executive domain | Dorsolateral prefrontal cortex | SFGdor |  | O | O |
|  |  | MFG |  | O | O |
|  | Ventrolateral prefrontal cortex |  |  |  |  |
|  |  | IFGoperc |  | O | O |
|  |  | IFGtriang |  | O | O |
|  |  | ORBinf |  | O | O |
|  | Insula | INS |  | O | O |
|  | Subcortical deep nuclei | AMYG |  | O | O |
| Memory domain | Limbic system | HIP |  | O | O |
|  |  | PHG |  | O | O |
|  |  | AMYG |  | O | O |
|  |  | PCG |  | O | O |
|  | Retrosplenial cortex | LING |  | O | O |
| Language domain | Wernicke area | SMG. L |  | O | - |
|  |  | ANG. L |  | O | - |
|  |  | STG. L |  | O | - |
|  |  | MTG. L |  | O | - |
|  |  | TPOmid. L |  | O | - |
|  | Broca area |  |  |  |  |
|  |  | IFGoperc. L |  | O | - |
|  |  | IFGtriang. L |  | O | - |
| Visuospatial domain |  | SOG |  | O | O |
|  |  | SPG |  | O | O |

A total of fifty-three Spearman partial correlation analyses were performed. Interhemispheric correlations were not investigated. Coefficients whose *p* value < 0.00094 (Bonferroni correction threshold: < 0.05/53) were selected for further analyses.
